# Facial Features: What Women Perceive as Attractive and What Men Consider Attractive

**DOI:** 10.1371/journal.pone.0132979

**Published:** 2015-07-10

**Authors:** José Antonio Muñoz-Reyes, Marta Iglesias-Julios, Miguel Pita, Enrique Turiegano

**Affiliations:** 1 Centro de Estudios Avanzados, Universidad de Playa Ancha, Viña del Mar, Chile; 2 Departamento de Biología, Universidad Autónoma de Madrid, Madrid, Spain; 3 Champalimaud Neuroscience Programme, Champalimaud Centre for the Unknown, Lisbon, Portugal; Brock University, CANADA

## Abstract

Attractiveness plays an important role in social exchange and in the ability to attract potential mates, especially for women. Several facial traits have been described as reliable indicators of attractiveness in women, but very few studies consider the influence of several measurements simultaneously. In addition, most studies consider just one of two assessments to directly measure attractiveness: either self-evaluation or men's ratings. We explored the relationship between these two estimators of attractiveness and a set of facial traits in a sample of 266 young Spanish women. These traits are: facial fluctuating asymmetry, facial averageness, facial sexual dimorphism, and facial maturity. We made use of the advantage of having recently developed methodologies that enabled us to measure these variables in real faces. We also controlled for three other widely used variables: age, body mass index and waist-to-hip ratio. The inclusion of many different variables allowed us to detect any possible interaction between the features described that could affect attractiveness perception. Our results show that facial fluctuating asymmetry is related both to self-perceived and male-rated attractiveness. Other facial traits are related only to one direct attractiveness measurement: facial averageness and facial maturity only affect men's ratings. Unmodified faces are closer to natural stimuli than are manipulated photographs, and therefore our results support the importance of employing unmodified faces to analyse the factors affecting attractiveness. We also discuss the relatively low equivalence between self-perceived and male-rated attractiveness and how various anthropometric traits are relevant to them in different ways. Finally, we highlight the need to perform integrated-variable studies to fully understand female attractiveness.

## Introduction

What makes a person attractive? This could be one of the most addressed questions in a broad range of fields in the social and natural sciences. The question is interesting beyond personal considerations, given the importance of attractiveness in human social interactions [1, 2]. Attractiveness often places attractive people in advantageous positions [3, 4]. For example, attractiveness can lead to both men and women receiving higher tips [5], benefitting from leniency bias in court decisions [6], and achieving higher long-term socioeconomic status [7]. In fact, evidence shows that our species tends to see attractive people as the embodiment of many positive qualities [1, 8–10]. This is the so-called “*what is beautiful is good*” stereotype [11].

Beauty is a complex construct that has been studied to a great extent throughout history [12]. It is commonly defined as an intrinsic property of some individuals that generates a number of emotions in those perceiving it [13]. Of these, pleasure is probably ubiquitous [12, 13]. Although pleasure elicited by the perception of beauty is not immediately linked to an expected utility [14], both beauty and its pleasurable effect in the opposite sex are relevant to the process of attracting potential mates [15, 16]. However, it is important to note that, regardless of sexual orientation, both sexes perceive male and female facial attractiveness similarly [17]. [39]

Several anthropometric traits have been associated with attractiveness [2, 18, 19]. Many of these features have been studied in detail in human faces, since the face is the most important part of the body in social interactions [20] and facial features are important determinants of overall attractiveness [21]. The facial features associated with attractiveness are: 1) Fluctuating asymmetry (FA; [22–25]), which measures the deviation from bilateral symmetry attributed to the individual, but not to the population [22, 26]; 2) facial averageness [27–30], which refers to the closeness of a face to the population average, 3) femininity of a female face [2, 15, 31, 32], which refers to the facial features that differentiate an individual as a woman; and 4) youthfulness [18, 32], which refers to the degree to which facial features relate to an early age. These features have been proposed as being linked to attractiveness, since they are indicators of desirable properties in the potential partners. For example, FA would be an indicator for developmental stability [33, 34], averageness for heterozygosity [35, 36] and both femininity [2, 37] and youthfulness [38–40] would signal fertility and health. The link of these features to fitness is well established, even when some alternative explanations have been proposed for their relation to attractiveness [16, 41–44].

In addition to facial features, features measured on the body, like the waist to hip ratio (WHR) [45] and the body mass index (BMI) [46], relate to attractiveness reliably. However, there is some controversy regarding the relative importance of these two [47–50], as both variables are usually correlated [46, 51]. In any case, both measurements are very often associated with attractiveness [47, 52–55], although there are some exceptions [56]. Women with a WHR of around .70 are classified as the most attractive by men of most cultures [47], and low values in BMI within the standard range, i.e., approximately 20, are typically regarded as more appealing [46, 51]. Values perceived as more attractive in both variables are usually related to better health [57–59] and higher fertility [60–63].

We investigated the link of a set of facial markers of female attractiveness to attractiveness in a group of young Spanish women. These were facial FA, facial averageness, facial sexual dimorphism (femininity) and facial maturity (youthfulness). We sought to measure how strongly these variables affect attractiveness when considered together. Furthermore, we controlled for age, WHR and BMI, as they have been described as influencing both measurements of attractiveness [53]. Hence, we evaluated simultaneously most of the key anthropometric traits in the study of women’s attractiveness described so far [2, 18, 15, 64, 65], since our main objective was to study the concurrent effect of all the variables, as they are perceived in real faces. Although it is very useful to isolate the effect of a single variable for the purpose of analysis, in real interactions it is impossible to separate the effect of different traits on the perception of attractiveness. Any attempt to fully understand how different features influence the perception of attractiveness should consider which trait, if any, is more influential, or whether their effects overlap or are independent. This would lead to an accurate assessment of the relevance of each variable to the perception of attractiveness. Furthermore, many of the studies previously mentioned evaluate the importance of the variables employing digitally manipulated faces (changing the studied features artificially) instead of using real faces. This calls into question the direct applicability of their findings to natural evaluations of real faces [2, 15, 18]. Therefore, a re-evaluation of the importance of said findings is necessary employing stimuli that are as close as possible to those that are present in real interaction.

Two methods have been previously employed to measure attractiveness. One measures women’s attractiveness according to male subjects' ratings [21, 47, 52, 53, 56]. The other method employs women’s perception of their own attractiveness [5, 53, 66–70]. Both variables are strongly related and show reciprocal influence, but they also show fundamental differences. Self-perceived and Male-rated scores correlate positively in several studies [53, 68], negatively in others [66] or do not correlate at all [69, 71]. For this reason, it has been proposed that both measurements of attractiveness should be treated as being related but independent [10], as in this study. Thus, we aimed to measure how strongly facial FA, facial averageness, facial maturity and facial sexual dimorphism affect both attractiveness evaluations, i.e., Self-perceived and Male-rated attractiveness, and to see to what extent these influences are similar.

When considering the variables individually, we expected to find a relationship in line with the results reported in previous studies, meaning that women with low facial FA, a younger facial appearance, and faces closer to the average and more feminine, will be perceived as more attractive both by men and by themselves. We also expected both measurements of attractiveness to correlate negatively with age. Additionally, we expected a woman with a WHR close to .70 and a BMI lower than 25 and higher than 19 to be considered attractive. We were not able to predict results when all variables are considered simultaneously, since all previous studies consider at most two variables concurrently [72–74]. Moreover, the different features have been measured in different ways, from morphometric computation to population ratings, and their effect on attractiveness has been estimated using different methods (real, constructed or manipulated images).

The three objectives of this work can be summarized as follows: 1) To study the simultaneous effect of variables previously studied separately to account for their relative importance and possible cross-effects; 2) To analyse the importance of these variables in a study with unmodified faces, which are a more natural stimulus; 3) To assess the effect of various facial features on Self-perceived and on Male-rated attractiveness. The differences are especially interesting given that the two variables are expected to show different effects on daily human interactions even when they are closely related.

## Materials and Methods

### Participants

The sample was composed of 266 female undergraduate students from the *Universidad Autónoma de Madrid* (Spain), ages 18 to 30 (21.60 ± 2.56). The number of participants had to be at least 215 to achieve a statistical power of .8, considering the previously calculated effect size of facial FA on facial attractiveness [22]. The participants received a payment of 10 € for their participation in the study.

### Ethics statement

The experimental procedures received the written consent of the *Comité de Ética de la Investigación* (CEI) (Research Ethics Committee) of the *Universidad Autónoma de Madrid* (Permit number: CEI-25-575). Each participant signed an informed consent form granting authorisation for the use of the data for research purposes.

### Anthropometric measurements

Facial Fluctuating Asymmetry, Facial Averageness, Facial Dimorphism (facial sexual dimorphism) and Facial Maturity were measured employing Geometric Morphometrics. Studying the shape using Geometric Morphometrics provides the advantage that the geometric information contained in the original shape is retained during the analysis [75–78]. The method employs a number of landmark coordinates that are placed directly on the face, rather than distances or angles, avoiding some of the well-documented problems of traditional morphometry: inconsistencies in the size correction methods followed, the lack of homology between measurements or issues with the relative positions of the traits employed as references [75, 78, 79]. Additionally, its statistical properties have proven to be better for the analysis of facial shape than those of traditional methods [80–81].

We measured Facial Fluctuating Asymmetry (Facial FA) following a previously described procedure [82] that has been successfully replicated [83, 84]. Participants were prompted to look straight into the camera (Nikon D90) with a neutral expression. To obtain the pictures, we used standardised conditions of light and head orientation (i.e., a frontal position). Facial FA was calculated from 39 facial landmarks that were identified on each face (for details see [82]) using the TPS software toolkit (available in http://www.life.bio.sunysb.edu/morph). Each author placed landmarks independently (i.e., they were placed four times) allowing the software to correct errors in landmark placement. The MorphoJ software [85] (available in http://www.flywings.org.uk/MorphoJ_page.htm) was used to calculate facial symmetry from the Procrustes distances between each landmark and the corresponding landmark of its mirror image. These distances were decomposed into directional asymmetry and FA by means of the Procrustes ANOVA method [86, 87]. As a measurement of FA we employed the Mahalanobis distance [88]. The error in landmark positioning was not significant (Procrustes ANOVA, error SS = 7.015 × 10^−2^, df = 59052, F = 0.108, p = 0.999).

We measured Facial Averageness by calculating the Procrustes distance between the landmark distribution of each participant’s average face, determined as described below, and a reference face [82, 88, 89]. We understand this feature as the difference in shape between an individual face and a prototype average face their socialmedia. ions with ins with ibut there strives fundamental differences among them Self-perceived is basically a learni(the reference face, see below), in line with other authors in different contexts [37, 90–92]. Each participant’s average face was computed from two of her photos and their mirror images to reduce the possible effects on each of these measurements of both landmark placement imprecision and the asymmetry of the particular faces. The reference female shape was obtained by averaging 358 images of self-reported White female students and their mirror images. All of the photographs employed in addition to the participants’ images to compute this and the other morphometric measurements were obtained from previously published and unpublished experiments [82, 84, 88, 89, 93]. Note that, in the case of this variable, lower scores correspond to higher averageness.

Facial Dimorphism and Facial Maturity were measured comparing the faces of the participants with, respectively, a set of male and girls’ photos in order to obtain a measurement that differentiated masculine from feminine faces (Facial Dimorphism) and adult from childlike female faces (Facial Maturity). We performed a PCA on the face shapes by using Geometric Morphometrics and then we included the significant factors (which account for at least 1% of variance) in a posterior discriminant analysis. These discriminant scores are employed as a measurement of Facial Dimorphism when male faces are included in the analysis [92, 94] and as a measurement of Facial Maturity when girls’ photos are included. We employed 320 facial images of self-reported White male students and 62 images of self-reported White schoolgirls aged between 11 and 14 years for this purpose. Male and girls faces were also averaged (using the sum of each image and its mirror images) to reduce any effect of differences in asymmetry in these measurements. The landmarks were the same employed in previous analyses. MorphoJ [85] allowed us to superimpose the shapes with a generalized least-square Procrustes fit. The covariance matrix was computed from these data of variation among individuals, and the PCAs were carried out on it.

For Facial Dimorphism computation we chose the first eleven PCs, which together accounted for 91.71% of the variance in facial landmark configuration. The resulting discriminant function classified correctly 98.3% of the faces. The discriminant function scores constitute an index of Facial Dimorphism, with small scores corresponding to more masculine faces.

For Facial Maturity computation we chose the first thirteen PCs, which together accounted for 90.12% of the variance. The resulting discriminant function classified correctly 93.6% of the faces. We employed the discriminant function scores as a measurement of Facial Maturity. In the case of this variable, lower scores correspond to mature faces and higher values correspond to more childlike faces.

Waist-to-hip Ratio (WHR) was obtained by dividing waist perimeter by hip perimeter. The waist of participants was measured in the lower girth region of the natural waist, directly above the umbilicus. The hip was measured along the widest point of the gluteal region. During measurements, participants stood with their feet together, arms relaxed to their sides, breathing normally, and with their body weight uniformly distributed to minimise the error caused by clothing. To calculate the body mass Index (BMI), each participant’s weight and height was measured barefoot and without heavy clothing. Height was measured with a manual stadiometer; weight was measured with a digital balance. In consideration to the participants, all measurements used to calculate WHR and BMI were taken only once and privately by a female researcher.

### Direct attractiveness measurements

Self-perceived attractiveness: Each participant reported an estimation of their own physical attractiveness in a 1-to-7 Likert-type scale in which 1 was the lowest score and 7 the highest (“How physically attractive do you consider yourself?”/ *¿Cómo de físicamente atractiva te consideras*?”).

Male-rated attractiveness: A group of 44 heterosexual men (M±SD, age: 31.63±6.00) rated the attractiveness of the participants from their pictures in a Likert 10-point scale (0 = “not attractive at all”/ “*nada atractiva*” to 10 = “extremely attractive”/”*extremadamente atractiva*”). Black and white photos were used in an attempt to reduce the effect of colour information. These images were presented randomly in nine different sessions in which raters evaluated 35 photos or fewer to avoid saturation. Not all the raters participated in each session and, moreover, they were instructed not to rate a woman if they could identify her. Consequently, not all of the evaluators rated all of the 266 photographs. We constructed an attractiveness index for each picture, averaging collected ratings for each photo. Only 40 of the 266 female photographs were rated by each of the 44 evaluators, but the average numbers of ratings per photo was 30.25. The consistency of the ratings was ICC(p) = 0.926, which is large enough to consider the process to be reliable [95].

### Statistical Analysis

Facial FA and Facial Dimorphism were normally distributed, and Male-rated attractiveness, Facial Averageness, and Facial Maturity followed the normal distribution after log conversion. However, there were four other variables that did not fit the normal distribution: Self-perceived attractiveness, age, WHR, and BMI. We transformed all variables to Z scores to compare easily the effect of variables with different scales [96]. The full data are available as electronic supplementary material (S1 Dataset).

We employed non-parametric Spearman’s Rank correlations and linear regression models followed by an analysis of the normality of residuals. All final models selected satisfy the normality condition. Variables included in the final two models we report were chosen through an automatic stepwise selection process. As some of the variables had been shown previously to correlate [97], we included the tolerance values (all of the values larger than .75) in the regression models. Furthermore, the condition indexes are lower than 2, indicating few problems of collinearity among the variables included in the different models [98]. All of the analyses described above were performed with the SPSS 15.0 package. To compare the effect of Facial FA on both Self-perceived attractiveness and Male-rated attractiveness, we computed two models including all of the variables with the exception of Facial FA. Then, we performed two simple regression models with the residuals of the models without Facial FA as the dependent variable and with Facial FA as the independent variable. Finally, we compared the coefficients obtained for Facial FA in these models following the method described by Zar [99].

## Results

Descriptive statistics of the analysed features and correlations with both measurements of attractiveness are included in Table 1. Correlation analyses showed that all anthropometric variables were associated with at least one of the two measurements of attractiveness (i.e., Self-perceived or Male-rated), except for Facial Dimorphism (See Table 1). There was a positive but weak relationship between Self-perceived and Men-rated attractiveness (Table 1). As some of the variables were significantly correlated, we computed the tolerance values of these variables in further regression analyses.

**Table 1:** Descriptive values of all variables, and Spearman’s Rank correlation between Self-perceived and Male-rated attractiveness with anthropometric traits.

	SPA	MRA	Facial Dimorphism	Facial Maturity	Facial Averageness	Facial FA	Age	WHR	BMI
**M±SD**	4.35 ± 1.06	3.67 ± 1.01	2.29 ±1.10	-0.56±0.99	5.94±.1.51 x 10^−2^	3.02 ±.36	21.60 ± 2.56	.724±.051	22.82 ± 3.89
**Correlations**	**SPA**	*rs* = .161**	*rs* = -.030	*rs* = -.018	*rs* = -.049	*rs* = -.142*	rs = .100	rs = -.225***	rs = -.208***
		**MRA**	*rs* = .049	*rs* = .176**	*rs* = -.251***	*rs* = -.093	rs = -.177**	rs = -.228***	rs = -.451***
			**Facial Dimorphism**	*rs* = -.274***	*rs* = -.016	*rs* = -.018	rs = -.124*	rs = .010	rs = -.102
				**Facial Maturity**	*rs* = -.194**	*rs* = .038	rs = -.079	rs = .092	rs = -.047
					**Facial Averageness**	*rs* = -.033	rs = .030	rs = -.007	rs = .119
						**Facial FA**	rs = -.009	rs = .199***	rs = -.044
							**Age**	rs = .043	rs = .146*
								**WHR**	rs = .191**

SPA = Self-Perceived Attractiveness, MRA = Male-Rated Attractiveness, FA = Fluctuating Asymmetry, BMI = Body mass index, WHR = Waist-to-hip Ratio.

*p < .05,

**p < .01,

***p < .001.

To analyse the effect of the different variables on Self-perceived and Male-rated attractiveness, we performed a series of linear regressions including all the anthropometric variables. First, we analysed the relationship of Self-perceived attractiveness to the different facial features (see Table 2), controlling for age, BMI and WHR. We performed backward and forward stepwise regression tests, ultimately choosing a model including Facial FA and the three control variables (BMI, WHR, and age). Facial FA and the BMI and WHR control variables were negatively associated with Self-perceived attractiveness. Age was positively associated, showing that older participants perceived themselves as being more attractive than younger ones. From this model, we tested the interactions between Facial FA and the control variables. None of these interactions were significant.

**Table 2:** Different models obtained from linear regression with enter and stepwise methods of Self-perceived attractiveness with facial measurements of averageness, dimorphism, maturity, Facial FA and controlling for age, BMI and WHR.

Method	R^2^ values	Variables	*Beta* value	*Tolerance*
Enter		Facial Dimorphism	-.114^+^	.847
		Facial FA	-.125*	.961
		Facial Maturity	-.050	.815
	.160	Facial Averageness	-.051	.926
		Age	.147*	.960
		BMI	-.327***	.777
		WHR	-.125*	.800
Stepwise		Facial FA	-.124*	.964
	.155	Age	.135*	.975
		BMI	-.315***	.815
		WHR	-.130*	.804

Note: R^2^ is corrected for the number of independent variables in the model.

*p < .05,

**p < .01,

***p < .001,

^+^p < .1.

Facial Averageness and Facial Maturity are log transformed. FA (Fluctuating Asymmetry); BMI (Body mass index); WHR (Waist to hip ratio).

Regarding Male-rated attractiveness, we also generated a model including the four morphometric variables and the three control variables (Table 3). The model finally chosen after both back and forward stepwise regression (see Table 3) included Facial FA, Facial Averageness and Facial Maturity. Of the control variables, only BMI remained. The variables included were negatively associated with this measurement of attractiveness, with the exception of Facial Maturity. This means that faces closer to the average and with low values for maturity and asymmetry were highly rated. To test the interactions between morphometric and control variables, we included the three control variables in the model. None of the interactions between morphometric variables and the control variables yielded a significant result.

**Table 3:** Different models obtained from linear regression with enter and stepwise methods, of Male-rated attractiveness with facial measurements of averageness, dimorphism, maturity, Facial FA and controlling for age, BMI and WHR.

Method	R^2^ values	Variables	*Beta* value	*Tolerance*
Enter		Facial Maturity	.239***	.815
		Facial Averageness	-.204***	.926
		Facial FA	-.090^+^	.961
	.296	Facial Dimorphism	.073	.847
		Age	-.087^+^	.960
		BMI	-.377***	.777
		WHR	-.092	.800
Stepwise		Facial Maturity	.211***	.943
	.286	Facial Averageness	-.203***	.929
		Facial FA	-.106*	.993
		BMI	-.437***	.972

Note: R^2^ is corrected for the number of independent variables in the model.

*p < .05,

**p < .01,

***p < .001,

^+^p < .1.

Male-rated Attractiveness, Facial Averageness and Facial Maturity are log-transformed.

Since Facial FA was shown to have an effect on both Self-perceived attractiveness and Male-rated attractiveness, we were interested in comparing the strength of the effect on each model. To that end, first we computed the residuals after adjusting both final models for all of the variables except for Facial FA. After this, we computed a simple regression model with the residuals as dependent variables and Facial FA as the independent variable (residuals Male-rated attractiveness and Facial FA: R^2^ = .017, beta = -.130, p < .05; residuals Self-perceived attractiveness and Facial FA: R^2^ = .015, beta = -.124, p < .05) and compared the betas. We did not find differences in the betas (t_(528)_ = .065, p = .948). Thus, Facial FA has a similar effect in the perception of attractiveness assessed in both Self-perceived and Male-rated tests.

## Discussion

The present study explores the effect of several facial features on two direct measurements of attractiveness: women’s self-perception and men’s rating of women's attractiveness. Our results show that these facial traits independently influence these two types of attractiveness measurements when real faces are employed. Facial FA affects both estimations of attractiveness with a similar intensity. However, while no other facial feature significantly influences Self-perceived attractiveness, Male-rated attractiveness is importantly affected by both Facial Maturity and Facial Averageness. This supports, at least partially, the hypothesis that self-perceived and third-party measurements of attractiveness are independent variables [10].

It might seem remarkable that Facial FA shows an equivalent effect on the two direct measurements of attractiveness, particularly considering that they are also influenced by different facial features. This observation confirms the importance of Facial FA in determining attractiveness. On the other hand, it may not be particularly surprising, given that symmetry is extensively considered a sign of attractiveness [10, 18, 19]. It is also an important factor in mating success in many species [34, 100]. To our knowledge, the effect of FA has never before been studied simultaneously on self-assessed and externally assessed attractiveness. The link between attractiveness and FA is usually attributed to the role of the latter as a reliable indicator of developmental stability [2]. However, there is also evidence that the preference for symmetrical partners is not necessarily related to potential mating benefits [42, 101], although this issue is under debate [102]. In any case, our results support past studies and confirm the inverse relationship between Facial FA and attractiveness [23, 25]. Our results are also consistent with a recent meta-analysis that finds a significant albeit weak link between the two [22]. Surprisingly, studies employing real faces that considered FA together with a second morphometric variable, like averageness [73] or masculinity [74], showed that FA was irrelevant in determining female attractiveness than the other variable. One possible explanation for this discrepancy might be an unexpected influence of uncontrolled variables that could be masking the true effect of FA. There are also procedural differences between these studies and ours that might provide an explanation. One of the studies followed traditional morphometry procedures [72], and presented, as described before, some of the usual problems associated with these procedures [75, 78, 79], like the variations in the method followed to standardize. The other studies [73, 74] employ Geometric Morphometrics, but the landmarks chosen are not exactly the same as ours. However, the main difference between our study and others is the number of participants: 266 women participated in our study, compared to 62 in [72], 48 in [73] and 112 in [74]. When one considers an expected effect size of Facial FA on facial attractiveness of .19 (according to [22]), together with the desired power of 80% and p-values lower than .05, one concludes that the sample size must be larger than 215.

More interesting is the influence of Facial Maturity and Facial Averageness on the attractiveness evaluations. The former variable measures the dissimilarity of the female participants’ faces to the faces of girls aged 11–14. The latter measures the closeness of the participants’ faces to the average female face. According to our results, Facial Maturity affects women’s attractiveness as rated by men, suggesting that men focus on cues for youth when evaluating female faces. This is in line with previous results showing that for men, the most attractive versions of adult women’s faces are those proportioned like young faces [103]. This result is also consistent with the widely described tendency of men to prefer younger women [5, 38, 104, 105]. It is likely that, in showing a preference for younger faces (i.e., a faces with fewer signs of maturity), men obtain highly reliable information about women’s reproductive value, fertility, and heritable disease resistance [40].

A higher Facial Averageness score (lesser averageness) shows a negative effect on attractiveness as evaluated by men, meaning that men prefer faces with shapes closer to the population average. This variable has been previously associated with female attractiveness in other studies [15]. Many of these worked with modified images [106–108], and it was difficult to assess the importance of the variable in real interactions. Some studies employed real faces, usually measuring Facial Averageness considering evaluators’ ratings [35, 109]. These studies also found a clear relationship between Facial Averageness and such different properties as attractiveness and health. However, morphometric measurements of averageness performed with classical morphometric methods do not show a significant correlation with attractiveness (a meta-review in [15]). This result is usually attributed to an inaccuracy of classic morphometric methods that makes them unable to represent the shapes properly. Therefore we decided to employ Geometric Morphometrics to compute Facial Averageness, given that this method of defining the facial shape has been successful in determining shape differences in many other species [110–112]. Ours is not the first attempt to measure averageness in human species employing Geometric Morphometrics [73]. We obtained similar results regarding Facial Averageness, but with a larger sample and considering more facial features in the analyses [73]. Moreover, we also replicated previous results showing a reduced importance for FA as compared to Facial Averageness employing both modified [108] and unmodified faces [72, 73].

Facial Dimorphism does not show a significant influence on any of the attractiveness measurements. In contrast, previous studies usually performed using measurements of attractiveness and femininity as evaluated by a third party [15], support this association. However, a reduction in the importance of femininity on attractiveness is observed when some variables are considered simultaneously in the analyses [113]. This result is interesting when one considers the effect of Facial Maturity on Male-rated attractiveness. These two variables, Facial Maturity and Facial Dimorphism, are expected to correlate strongly, since adult female faces are more similar to children’s faces than are male faces [18]. However, Facial Maturity has an effect on Male-rated attractiveness, while Facial Dimorphism does not. Moreover, in our study these two variables correlate negatively. This is not surprising, because although during adolescence male faces change more drastically than female faces [18], there are still many traits in a woman’s face that change during this period, under the influence of sex hormones [114]. These traits differentiate adult women’s faces from those of young girls, especially lip thickness [72, 115], pronounced cheekbones and narrow cheeks [31, 116, 117]. Hence, such secondary characteristics are the features that draw a clear line between a feminine face and an immature face, that is, between Facial Dimorphism and Facial Maturity. Given that our measurement method maximizes the differences between groups, it is clear that Facial Dimorphism considers mainly those feminine features unrelated to a childlike appearance (given that those two correlate negatively). This suggests that, when rating females' facial attractiveness, men attach less importance to facial features that distinguish a female from a male face (e.g., thickening of the lips), than to facial features signalling youth. This particular analysis has been possible because our Facial Dimorphism measurement is not significantly affected by childlike features. Therefore, our measurement procedure allows us to discriminate between feminine features related to the retainment of childlike features from those features specifically developed in women, a distinction that usual measurements of femininity do not make [74, 94]. It is remarkable that this preference for youth indicators over sexual dimorphic features is in contrast with the widely known tendency of men to prefer female bodies with a clear feminine shape [80–82, 86, 91]. However, it is important to note that, for the most part, sexual dimorphism and sexual maturity are indicated by the same features in the female body.

Our results replicate previous studies [5, 53] that demonstrate that body traits (BMI and WHR) affect the self-perception of attractiveness. This confirms the results from studies that considered these traits separately [46, 47], and to a certain extent, those that studied them simultaneously [48, 50, 51, 63]. According to a previous study [118], BMI, as does the percentage of body fat, has an effect on Male-rated facial attractiveness, but WHR does not. It is noteworthy that facial adiposity is predicted more precisely by BMI than by WHR [54, 119], and that facial adiposity is negatively associated with health [54, 120]. Therefore, the effect of BMI on the facial rating of attractiveness by men might be understood as a factor affecting men’s perception of women’s health, based on facial adiposity. The lack of association between WHR and Male-rated attractiveness can be explained by the fact that male raters did not observe the body of participants, whereas it stands to reason that women consider all their body traits in their self-perception. However, several past studies have shown that signs of attractiveness are correlated [31, 121–123] (but see [124]), and, specifically, that women's faces and bodies both influence attractiveness scores strongly [56]. Therefore, we would expect variables affecting body attractiveness, like WHR, to affect facial attractiveness as well. However, we are not able to detect this relationship, which stresses the necessity for further study of the correlation between the variables related to attractiveness.

Finally, age also shows an effect on Self-perceived attractiveness. However, in contrast with the literature [38, 105], we found a positive association between the two. This apparently counter-intuitive result can be explained by considering that our participants were a homogeneous sample of very young women (see Table 1). Therefore, the positive effect of age on attractiveness may be due to the particular age of the older women in our sample, who are closer to their sexual maturity and fecundity peaks [125, 126].

Interestingly, we find a weak relationship between Self-perceived attractiveness and Male-rated attractiveness (Table 1), which has also been reported in previous studies [53, 68, 69] (though others do not find this relationship [66, 71]). In fact, some authors have proposed that these two measurements should be considered to be completely different variables [10]. Such a suggestion is supported by our results, as different morphometric variables have a different effect on each of these two measurements of attractiveness. This is not surprising, given that the latter show fundamental differences. Male-rated attractiveness depends basically on physical features (particularly if the rating is based on photos). On the other hand, Self-perceived attractiveness is a feature built in the course of an individual’s life, and it is based at least partially on the individual’s interactions with their social environment. Therefore, considering that Facial FA is not expected to change significantly after puberty [127, 128], its effect on Self-perceived attractiveness is not a surprising result, given that it has had a longer time than other variables to establish itself as an attractive or unattractive feature for other people. However, Facial Averageness, which is equally stable, has failed to show an effect on Self-Perceived attractiveness. These results confirm the difficulty of making clear predictions on the combined effect of variables not studied together before.

As a general conclusion, and in accordance with our results, we suggest that future studies on attractiveness should measure the importance of any morphometric variable in real faces, given that currently there are enough morphometric tools to properly measure it. We recommend a cautious use of modified images, given that some variables render different results when artificial, computer manipulated images are used, potentially introducing confusion to the scientific discussion (for a review of the differences between results with modified and non-modified images see [18]). We also recommend analysing the effect of different variables simultaneously instead of one by one, since the inclusion of some variables reduces considerably the significance of others. Similarly, we suggest to include in any experimental design the self-perceived attractiveness score in addition to the attractiveness scores from third-party raters, because the two are distinctively different variables and because the former is likely to have greater importance on any conscious effect of attractiveness on individual behaviour.

Finally, we want to point out that the present study, like many others in the field, was carried out exclusively with a university population of self-described White women from a western culture. For this reason, before generalizing our findings to the rest of the human species, it would be necessary to extend the analysis to a greater range of ages, socio-cultural strata and ethnic groups. This is especially important considering that many of the analysed variables have shown different importance in different societies [32, 47, 129].

## Supporting Information

S1 Dataset Complete facial features dataset. The dataset includes all the variables considered and information about the computing process for Facial Maturity, Facial Dimorphism and Male-rated Attractiveness.
(XLSX)
